# CAF-1 in Replication and Repair: A Critical Genetic Mediator of Synthesis-Coupled Nucleosome Assembly

**DOI:** 10.3390/biom16091277

**Published:** 2026-09-03

**Authors:** Ian Hall, Carly A. Nowoj, Lynne M. Dieckman

**Affiliations:** 1 Department of Chemistry and Biochemistry, Creighton University, Omaha, NE 68178, USA; 2 Program in Chemistry, University of Arkansas at Little Rock, Little Rock, AR 72204, USA

**Keywords:** chromatin assembly factor 1 (CAF-1), proliferating cell nuclear antigen (PCNA), replication-coupled nucleosome assembly, repair-coupled nucleosome assembly

## Abstract

Eukaryotic genomes are organized into chromatin, a highly compact structure in which DNA is packaged into nucleosomes. Nucleosome formation, where DNA is wrapped around histone proteins, is essential for genome stability. This compaction protects DNA from damage and regulates accessibility of genes. Nucleosomes must be disassembled and reassembled during DNA replication and repair. These processes require precise regulation of histone folding, transfer, and deposition by a diverse network of histone chaperones. Chromatin assembly factor 1 (CAF-1) is a conserved histone chaperone that specifically deposits newly synthesized histones during replication-coupled and repair-coupled nucleosome assembly. The sliding clamp proliferating cell nuclear antigen (PCNA) serves as a regulatory scaffold during these processes by recruiting CAF-1 and many other proteins to sites of DNA replication and repair. Recent structural and biochemical studies have revealed increasingly complex mechanisms underlying PCNA-mediated CAF-1 recruitment, involving multiple protein interaction motifs, DNA-binding domains, and regulatory mechanisms that ensure efficient nucleosome assembly. This review summarizes current advances in understanding the molecular mechanisms by which human and yeast CAF-1 complexes are recruited to sites of DNA synthesis and how CAF-1 function is coordinated with other histone chaperones during replication and repair. These studies have provided important insights into how cells coordinate DNA metabolism with epigenome maintenance to preserve genome integrity.

## 1. Introduction

The expansive genomes of eukaryotes are maintained in chromatin, a highly compact nucleoprotein complex. The minimal structural unit of chromatin is the nucleosome, which is comprised of approximately 147 base pairs of DNA spooled around an octamer of histone proteins [1]. The full set of histone proteins present in the nucleosome includes two copies each of H2A, H2B, H3, and H4. In the core of the nucleosome, two H3-H4 heterodimers assembled into a (H3-H4)_2_ tetramer that binds to and organizes the central 70–80 base pairs of nucleosomal DNA [1,2]. The periphery of the nucleosome is completed by two flanking H2A-H2B heterodimers that bind an additional 30–40 base pairs of DNA [1].

The binding of histones to DNA is driven by favorable electrostatic interactions between the basic, positively charged amino acid residues on histones and the negatively charged, acidic phosphate backbone of DNA. Histone–DNA interactions occur in a non-specific and sequence-independent manner. Accordingly, histone–DNA interactions are tightly regulated to ensure proper nucleosome assembly. Histone chaperones are highly acidic proteins that interact with and block the basic DNA-binding surfaces of histones to prevent non-specific association of histones with DNA outside of nucleosome assembly [3,4]. Histone chaperones usually specialize in binding the canonical histone dimers H2A-H2B or H3-H4, while some histone chaperones have specificity for histone variants [5].

The compaction of eukaryotic DNA into nucleosomes promotes genome stability by sequestering DNA against histones, thereby limiting solvent accessibility and shielding the DNA from endogenous and exogenous sources of damage. However, the DNA in nucleosomes is also inaccessible to the cellular machineries required for DNA-dependent processes like replication and repair. To facilitate DNA replication and repair, nucleosomes are temporarily disassembled to allow the passage of the replication fork or the repair machinery [6]. Nucleosome reassembly is tightly coupled to these processes to maintain genomic integrity. During replication-coupled nucleosome assembly (RplNA) and repair-coupled nucleosome assembly (RprNA), histones are deposited onto DNA by a diverse network of histone chaperones and accessory proteins [5,6].

The eukaryotic sliding clamp, proliferating cell nuclear antigen (PCNA), is a homotrimeric processivity factor that encircles DNA and recruits a myriad of protein-binding partners to DNA during almost all DNA-templated processes [7]. During nucleosome assembly, PCNA does not directly interact with histones. Instead, PCNA functions as a molecular scaffold that regulates nucleosome assembly by recruiting histone–histone chaperone complexes to DNA and promoting histone deposition [7,8]. The molecular mechanisms that govern PCNA-mediated recruitment of specific histone chaperones to DNA during RplNA and RprNA remains a highly active and critical area of epigenetic research.

Recent structural and biochemical studies have significantly refined our understanding of PCNA-mediated, de novo nucleosome assembly by chromatin assembly factor 1 (CAF-1) during both RplNA and RprNA. CAF-1 is a conserved heterotrimeric protein complex that is most widely studied in humans and budding yeast. CAF-1 specifically binds nascent H3-H4 dimers and deposits them onto DNA while the recycling of parental histones is facilitated by other chaperones [9,10]. CAF-1 is composed of three distinct subunits that are conventionally categorized by molecular weight: the large subunit (human p150/yeast Cac1), the middle subunit (human p60/yeast Cac2), and the small subunit (human p48/yeast Cac3) [11,12]. This article reviews PCNA- and DNA-mediated mechanisms by which human and yeast CAF-1 are recruited to sites of nucleosome assembly during DNA replication and repair.

## 2. PCNA Architecture and Mechanisms of CAF-1 Recruitment

CAF-1 is the histone chaperone responsible for deposition of nascent H3 and H4 histones onto newly synthesized DNA during nucleosome assembly, coordinating chromatin restoration with DNA replication across the genome [13,14,15]. The function of CAF-1 is coupled to DNA replication and repair through its interaction with the DNA sliding clamp, PCNA [8]. Accordingly, CAF-1 recruitment and function is critical in regions of the genome that are loaded with PCNA, such as regions undergoing DNA replication or synthesis-dependent DNA repair [16].

**Figure 1:**
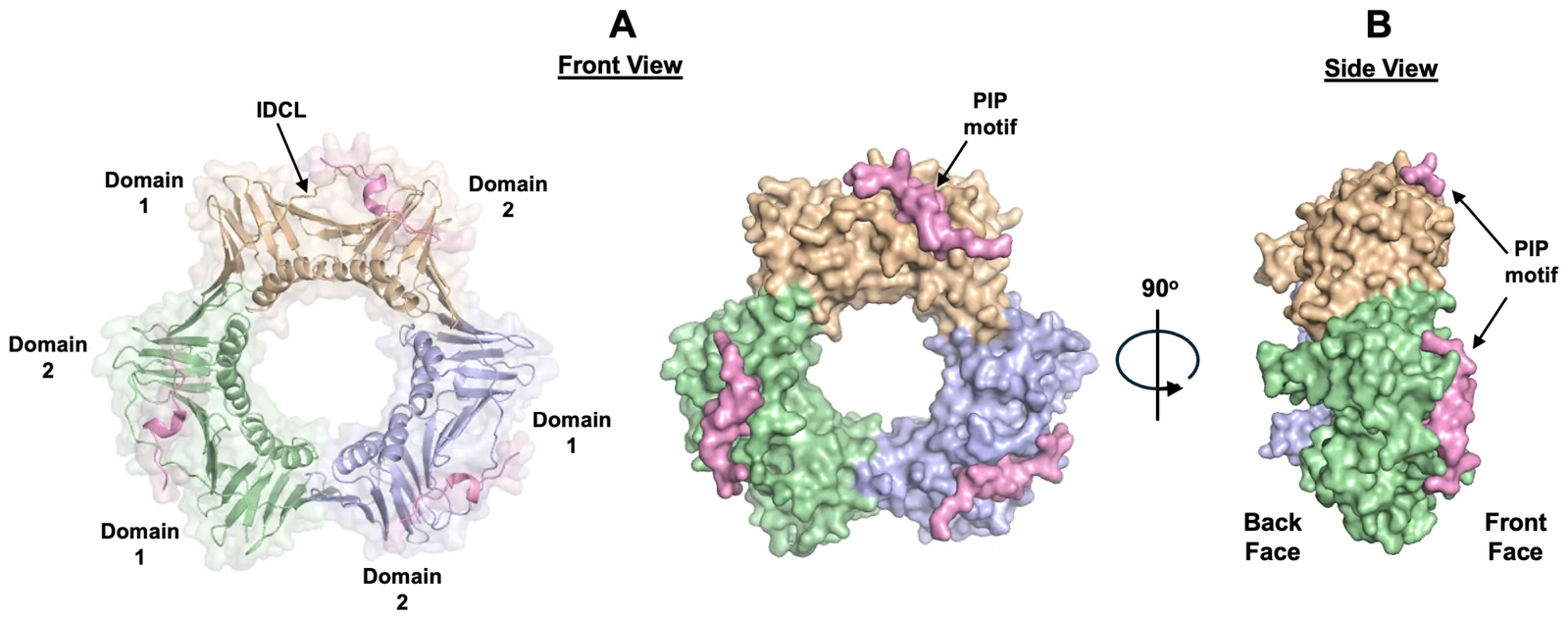
The structure of PCNA bound to a PIP motif (PDB 8THW). (**A**) Front view of the structure of a PCNA homotrimer bound to a PIP motif in each subunit, shown in both ribbon (left image) and surface (right image) representations. Each monomer of PCNA is shown in a different color and the orientation of each domain in PCNA is indicated. The location of the IDCL within the PCNA structure and the PIP-binding pocket bound to a PIP motif (pink) are shown. (**B**) Side view of the structure of PCNA bound to PIP motifs, shown as a surface representation. The PIP motifs of PCNA-interacting proteins bind to the front face of PCNA.

PCNA is a homotrimeric, ring-shaped protein that encircles duplex DNA and slides freely along it once loaded, serving as the central processivity and recruitment factor for a myriad of proteins involved in DNA metabolism [7,17]. Each monomer of PCNA consists of two similar domains [17]. Domain 1 (residues 1–117) is connected to domain 2 (residues 135–258) by a long, flexible interdomain connecting loop (IDCL; residues 118–134) (Figure 1). The three subunits adopt an alternating orientation, with domain 1 of one subunit interacting with domain 2 on the adjacent subunit. Twelve positively charged α helices, two from each domain, line the interior surface of the ring and allow negatively charged DNA to pass through. The central hole of PCNA has a diameter of approximately 35 Å, larger than the diameter of DNA (approximately 20 Å). The subunit interface is characterized by β-strands from domain 1 and domain 2 of adjacent subunits, forming an extended β-sheet.

PCNA consists of two structurally distinct faces, referred to as front and back faces. The front face points in the direction of DNA synthesis and contains the C-terminal ends of each monomer. The back face points away from DNA synthesis, toward the direction of nucleosome assembly. Most proteins that bind to PCNA do so through a conserved eight-residue motif called a PCNA-interacting peptide (PIP) motif (Figure 1). The canonical consensus sequence of a PIP motif includes a glutamine in position one, a hydrophobic residue in position four, and aromatic residues in positions seven and eight [18]. The PIP motif of PCNA-interacting proteins binds a hydrophobic pocket on the front face of PCNA near the IDCL [7]. The conserved glutamine in position one of the PIP motif inserts in the “Q pocket” of PCNA, and residues in positions four through eight form a short, 3_10_ helix that binds in a hydrophobic pocket near the IDCL [7].

**Figure 2:**
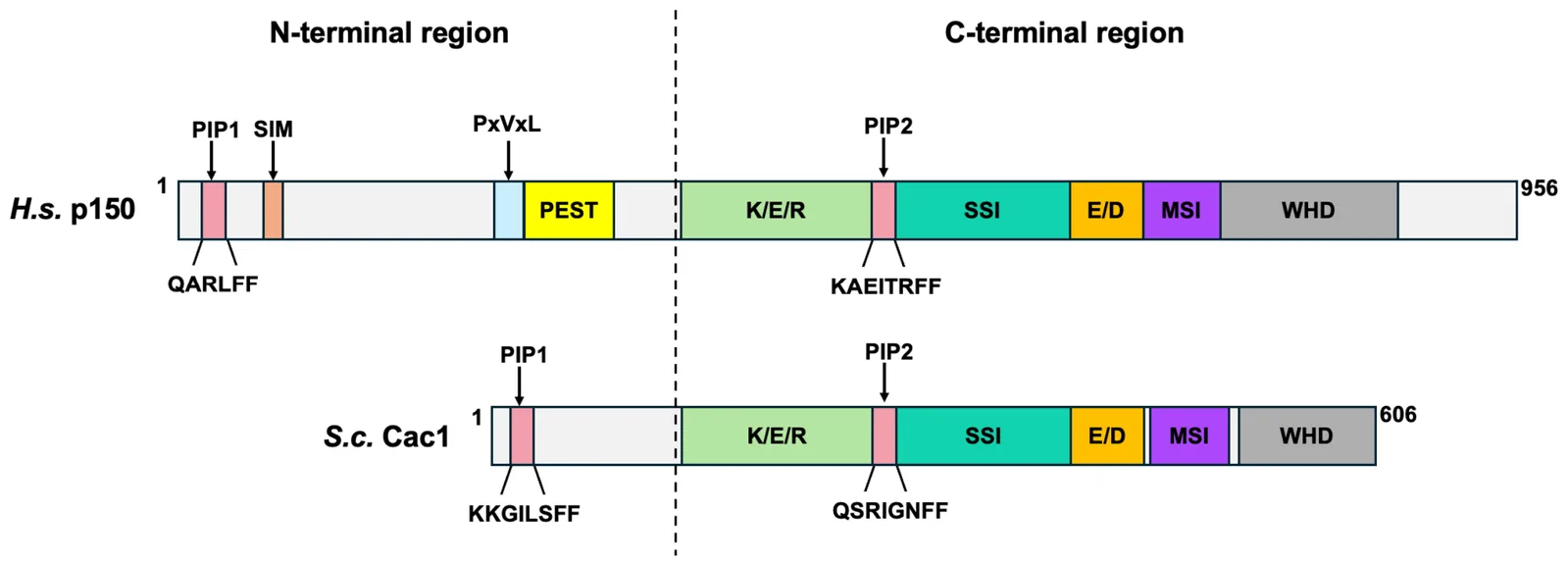
Domain organization of the large subunit of CAF-1 in humans and yeast. Linear architecture of the large CAF-1 subunit from *Homo sapiens* p150 (**top**) and *Saccharomyces cerevisiae* Cac1 (**bottom**) are shown. The motifs and domains in both the N-terminal region (**left**) and C-terminal region (**right**) of each protein are labeled. The same colors are used to indicate corresponding domains or regions in H.s. p150 and S.c. Cac1. PIP motif sequences are indicated beneath each protein.

Like other PCNA-interacting proteins, CAF-1 interacts with PCNA through a conserved PIP motif. Human CAF-1 contains two known PIP motifs on the p150 subunit, named PIP1 and PIP2, for their order within the protein sequence [19] (Figure 2). The PIP1 motif consists of a mostly canonical PIP sequence (containing only six residues but retaining all four of the conserved amino acids) and binds PCNA strongly in vitro but is not required for nucleosome assembly in vivo [19]. In contrast, the PIP2 motif, located in the C-terminal region of p150, represents a non-canonical motif, binds PCNA weakly, and is required for nucleosome assembly in vivo [19].

Several mutant forms of PCNA have been identified that selectively inhibit the ability of CAF-1 to bind PCNA in vitro and to promote gene silencing in vivo [20]. The structures of these mutant proteins were determined using X-ray crystallography, which revealed that a possible CAF-1 interaction site may exist in a surface cavity on the front face of PCNA near the PIP-binding pocket [21,22]. Further research is needed to confirm this novel interaction and to elucidate the role it plays in recruiting CAF-1 to sites of nucleosome assembly.

The structure of human PIP2 bound to PCNA was recently reported and suggests a mechanism of interaction divergent from the typical PIP–PCNA interaction [23]. Rather than relying on the canonical glutamine–hydrophobic–aromatic anchoring to the PIP-binding pocket, the human p150 PIP2 element was reported to engage PCNA through a non-canonical cation-π interaction. In this mechanism, an arginine in position six of the PIP2 motif inserts into the PIP-binding pocket of PCNA that is normally occupied by the aromatic anchor residues of canonical PIP motifs. This arginine engages a PCNA tyrosine through a cation-π interaction rather than through hydrophobic packing. As this is a novel interaction identified for PIP–PCNA complexes, future investigations will be valuable to determine whether this is the exclusive binding mode or if multiple interaction states exist. This is particularly relevant given that the underlying crystallographic data provided clear electron density for only three PIP2 residues: the arginine involved in the non-canonical interaction and the two conserved phenylalanine residues in positions seven and eight of the motif. Point mutation of the arginine residue to an aspartic acid disrupted both PCNA binding and CAF-1 chromatin assembly activity in vitro, establishing that this residue is functionally important regardless of the precise structural configuration.

Historically, yeast CAF-1 was believed to contain one PIP motif, in the C-terminal region of the Cac1 subunit [24,25]. Recently, an additional PIP motif was identified in the N-terminal region of Cac1, similar in location to the human PIP1 motif in p150 [26] (Figure 2). Thus, this motif is termed the PIP1 motif and the C-terminal PIP sequence is now termed the PIP2 motif. The structure of the PIP2 motif from Cac1 bound to PCNA was recently determined, which showed a largely canonical mode of interaction (PDB 8THW) [27] (Figure S2). As expected, the conserved hydrophobic PIP residues inserted into the PIP-binding pocket on the front face of PCNA. However, this structure also revealed an extended interaction surface beyond the core eight-residue PIP consensus—residues immediately N- and C-terminal to the motif make additional contacts with the C-terminus and IDCL of PCNA, respectively, a feature shared by only a small number of other characterized PIP–PCNA interactions [28,29,30]. Mutational analysis indicated that positively charged residues flanking the PIP motif contribute to the affinity of the interaction, while a negatively charged residue N-terminal to the core motif appears to limit PCNA affinity.

The presence of the additional, non-canonical PIP1 motif in CAF-1 makes this one of only a few known yeast proteins to utilize multiple PCNA interaction elements. These two motifs exhibit distinct binding kinetics and affinities for PCNA, suggesting specialized functions for each during nucleosome assembly [26]. Moreover, these studies showed the PIP1 motif contains an additional binding surface C-terminal to the motif that forms a β-sheet with the IDCL of PCNA (Figure S1). Similar interactions have not previously been observed in yeast PCNA-binding proteins, but only in a few cases of human proteins such as p21 and FEN1 [28,29]. Functional assays revealed that mutations within either of the yeast PIP1 or PIP2 motifs individually do not completely disrupt gene silencing in vivo, indicating possible redundancy between the two motifs. Disruption of the Cac1 PIP1 motif had no discernable impact on gene silencing, whereas a PIP2 mutation elicited a moderate silencing defect, suggesting the PIP2 motif may be more dominant in recruitment of CAF-1 to sites of nucleosome assembly. However, simultaneous disruption of both the PIP1 and PIP2 motifs completely abolished silencing to levels comparable to when the Cac1 gene was deleted, demonstrating both PIP motifs are together essential for in vivo CAF-1 function.

Human and yeast CAF-1 share structurally analogous dual-PIP motifs, yet the PIP1 and PIP2 motifs appear to have opposite relationships in sequence, PCNA-binding mechanisms, and functional roles between species [19,26,27]. In contrast to the functionally redundant yeast motifs, the human PIP2 motif is strictly critical for nucleosome assembly and the PIP1 motif is dispensable. Based on this evidence, we suggest the presence of both a canonical and a non-canonical motif in CAF-1 are essential for nucleosome assembly, and that the coordination of these two motifs enables CAF-1 to access the DNA behind the PCNA ring for histone deposition. Overall, these studies indicate that the sequence, length, binding kinetics, affinities, and structural variations within PIP motifs likely serve a critical role in regulating PCNA partner selectivity at the replication fork.

## 3. CAF-1 Architecture and Its Interactions

CAF-1 is a heterotrimeric complex that is conserved among eukaryotes and is composed of subunits categorized by molecular weight [5,11,12]. The large subunit is called p150 in humans (also denoted CHAF1A) and Cac1 in yeast. The intermediate or middle subunit is called p60 in humans (also denoted CHAF1B) and Cac2 in yeast. The small subunit is called p48 in humans (also denoted RBBP4) and Cac3 in yeast. The large subunit forms the structural backbone of the complex: it self-associates to create a dimerization interface, scaffolds the recruitment of the middle and small subunits, and carries most of the CAF-1 function required for RplNA and RprNA [15,31,32].

### 3.1. Large Subunit

The large subunit of CAF-1 is organized into an N-terminal region and a C-terminal region that differ substantially in both their domain content and their degree of conservation between yeast and human CAF-1 variants [5,11,31] (Figure 2). The N-terminal region contains one or more binding elements for other proteins, most of which are best characterized in humans. The N-terminal region is largely disordered in both species and consequently has had little structural characterization compared to the C-terminal region [31,32]. Additionally, the N-terminal region is dispensable for nucleosome assembly in vitro whereas the C-terminal region is strictly required [11,15,31]. The C-terminal region is more strongly conserved and is a scaffold for assembly of the CAF-1 heterotrimeric complex [15,31,32].

### 3.2. N-Terminal Region

The N-terminal region of p150 contains a PIP motif (PIP1), a SUMO-interacting motif (SIM), a heterochromatin protein 1 (HP1)-interacting PxVxL motif, and a PEST domain. The corresponding N-terminal region of Cac1 remains largely uncharacterized. Apart from the recently identified PIP1 motif, no additional functional elements have been defined in this region.

The role of the p150 SIM motif in CAF-1 function is not yet clear. Mutation of the SIM prevents association of p150 with nucleolar proteins and chromosomes, but the CAF-1–PCNA interaction is unaffected by this mutation [33]. This suggests a role for the SIM in regulating CAF-1-protein and CAF-1-DNA interactions in the nucleolus, but not with the CAF-1–PCNA interaction. The PxVxL motif is a pentapeptide recognition element used by numerous nuclear proteins to dock onto the chromo shadow domain of HP1 [34,35]. Biochemical and cell biology studies have shown that this interaction is important for two distinct aspects of p150 function: the stable retention of CAF-1 at heterochromatin outside of S phase, and the recruitment of the HP1α—an isoform of HP1—to sites of DNA damage [36,37]. Structural studies of the p150–HP1 complex established the molecular basis of this interaction, revealing that the PxVxL motif binds in an extended conformation within a conserved hydrophobic groove at the chromo shadow domain dimer interface [34,35]. The PEST domain (a sequence enriched in proline, glutamic acid, serine, and threonine) generally marks proteins for rapid proteasomal turnover [38]; however, the function of the p150 PEST domain has yet to be explicitly defined.

### 3.3. C-Terminal Region

The C-terminal region of the large subunit is more highly conserved between humans and yeast and contains the domains that mediate histone binding, DNA binding, subunit assembly, and nucleosome assembly [14,15,39]. The C-terminal region contains, in primary sequence order, a K/E/R domain, a second PIP motif (PIP2), a small subunit-interacting (SSI) domain, an E/D domain, a middle subunit-interacting (MSI) domain, and a winged helix domain (WHD) (Figure 2).

The K/E/R domain is a DNA-binding element [14,40]. The structure of the yeast K/E/R domain, named for the lysine-, glutamate-, and arginine-rich composition of the segment, was recently determined by X-ray crystallography (PDB 8DEI) [14]. Although previously predicted to form a coiled-coil motif, the crystal structure of the K/E/R domain supports a single alpha helix (SAH) motif. Interestingly, SAH motifs do not canonically bind DNA [41]. However, structural and biophysical characterization of the yeast K/E/R domain demonstrates that the domain selectively binds DNA fragments of at least 40 base pairs [14]. The selective DNA-binding activity of the K/E/R domain is functionally significant because CAF-1 preferentially binds lengths of DNA consistent with tetrasome formation (containing two H3-H4 dimers and ~70–80 base pairs of DNA) to deposit two dimers of H3-H4 [14,15,32].

Deletion of the K/E/R domain in yeast produces a mild phenotype, with retained competence for tetrasome assembly and only modest sensitivity to DNA-damaging agents or defects in heterochromatic silencing, indicating that the WHD (discussed below) can partially compensate for loss of K/E/R-mediated DNA binding [40]. Concurrent disruption of the K/E/R domain and WHD produces a much more severe phenotype, with pronounced defects in DNA damage survival and heterochromatin formation [40]. In both humans and yeast, the K/E/R domain is immediately proceeded by the PIP2 motif. This arrangement has been proposed to coordinate DNA and PCNA binding by CAF-1 [31,32]. Recent work using CAF-1 complexes and PCNA from the fission yeast *S. pombe* suggest that K/E/R domain-mediated DNA binding allosterically stimulates binding of PCNA by CAF-1 [42]. The conservation of this mechanism in humans and budding yeast has yet to be established.

**Figure 3:**
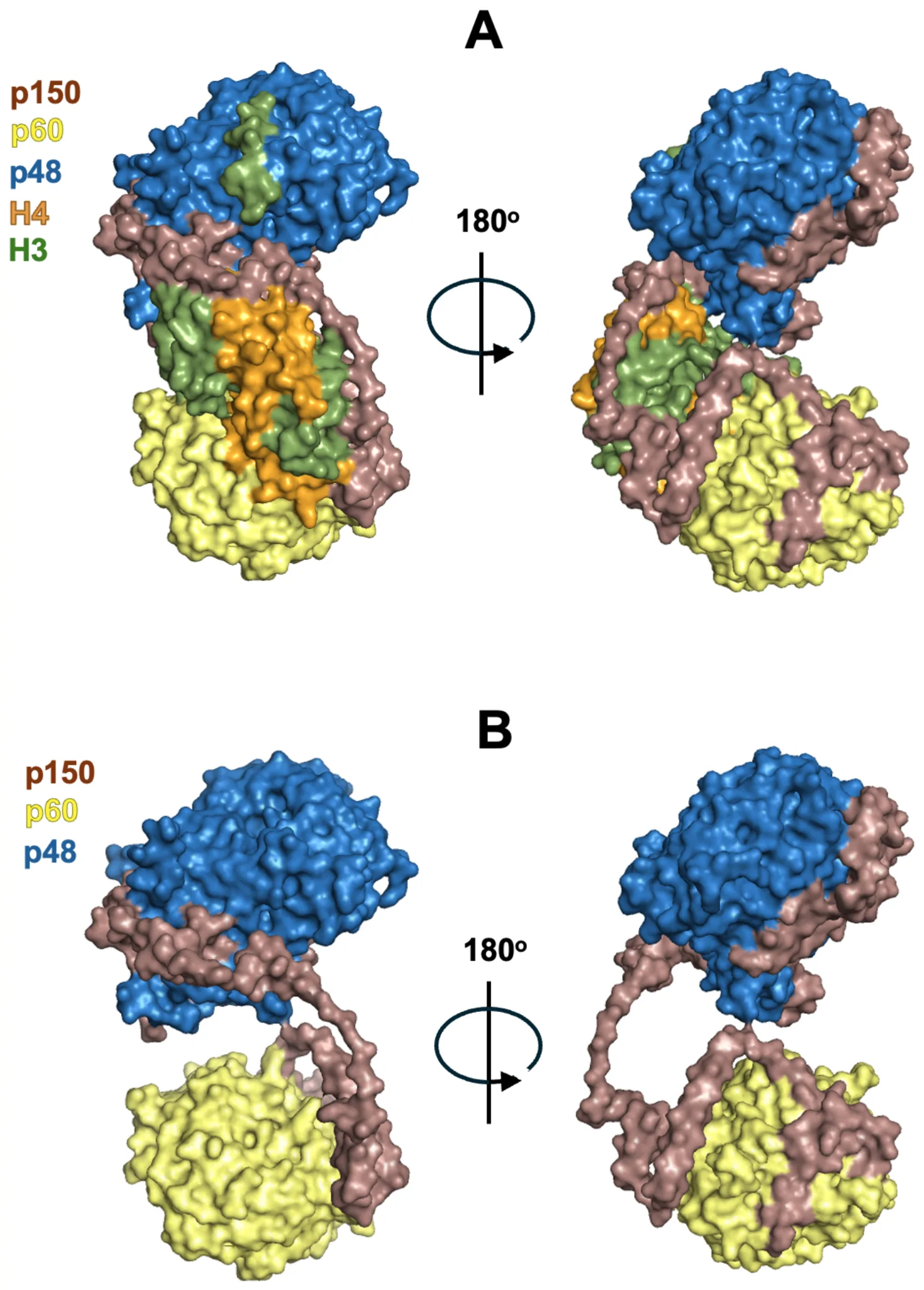
Structure of the human CAF-1-H3-H4 complex (PDB 8IQG). (**A**) The organization of the holo CAF-1-H3-H4 complex, with each CAF-1 subunit and histone protein labeled in a different color (p150 is mauve, p60 is yellow, p48 is blue, H4 is orange, and H3 is green). (**B**) The organization of the CAF-1 heterotrimer with histone proteins removed and histone-binding surfaces exposed.

The C-terminal SSI domain mediates the physical association between the large and small CAF-1 subunits [43]. The SSI domain directly binds the small subunit, but the small and middle subunits do not physically interact in the CAF-1 heterotrimeric complex. The SSI is immediately upstream of the E/D (acidic, glutamate/aspartate-rich) domain. The highly acidic E/D domain utilizes electrostatic interactions to engage the basic, positively charged surfaces of the H3–H4 histone dimer [32]. In the cryo-EM structure of the human CAF-1–H3-H4 complex, a single H3-H4 heterodimer is engaged with one CAF-1 heterotrimer predominantly through the middle subunit (p60) and the p150 E/D domain [32] (Figure 3). In the presence of DNA, two such CAF-1–H3-H4 units associate into a dimeric complex in which the two H3-H4 heterodimers are positioned for tetramer formation. Studies have shown a similar large subunit–small subunit histone binding mechanism occurs in yeast. Mass spectrometry experiments combined with biochemical assays supported the hypothesis that a H3-H4 interaction surface is only formed when Cac1 is pre-bound to Cac2 [43]. Here, the Cac1-bound Cac2 and the Cac1 E/D domain form a composite interface that together bind H3-H4.

Electron microscopy analysis revealed the overall architecture of the yeast CAF-1 complex adopts a bilobal shape [44]; however, a high-resolution structure of the full-length complex has not yet been determined. The structure of human CAF-1 bound to histones further demonstrated that CAF-1 facilitates the formation of tetrasomes in a right-handed orientation [32]. This geometry is opposite to the left-handed DNA wrapping in a mature nucleosome, suggesting CAF-1 generates a right-handed precursor that must undergo a handedness inversion. The structure further revealed that CAF-1 remains associated with the right-handed tetrasome intermediate, supporting a role for the chaperone in stabilizing this assembly intermediate prior to nucleosome maturation.

The MSI domain mediates the association between the large and the middle subunits [43]. Again, this association is independent of the large–small subunit interactions. Immediately adjacent to this domain is the WHD, a compact, helix–turn–helix DNA-binding domain [25]. The WHD constitutes the second DNA-binding element within CAF-1, alongside the K/E/R domain. The DNA-binding activity of the WHD is sequence-independent [25], consistent with the role of CAF-1 as a genome-wide, general nucleosome-assembly factor that acts at replication forks and repair sites of arbitrary sequence context. Point mutations in the yeast WHD that selectively abolish its DNA-binding activity but maintain its structure cause defects in transcriptional silencing at heterochromatic loci and increased sensitivity to DNA-damaging agents, demonstrating the importance of the WHD in genome maintenance [25]. In fission yeast, by contrast, the WHD retains the winged-helix structural fold characteristic of the domain but does not itself bind DNA, and yet remains functionally essential to CAF-1 function [42]. The functional and evolutionary significance of differences in the WHD among these organisms remains unclear.

**Figure 4:**
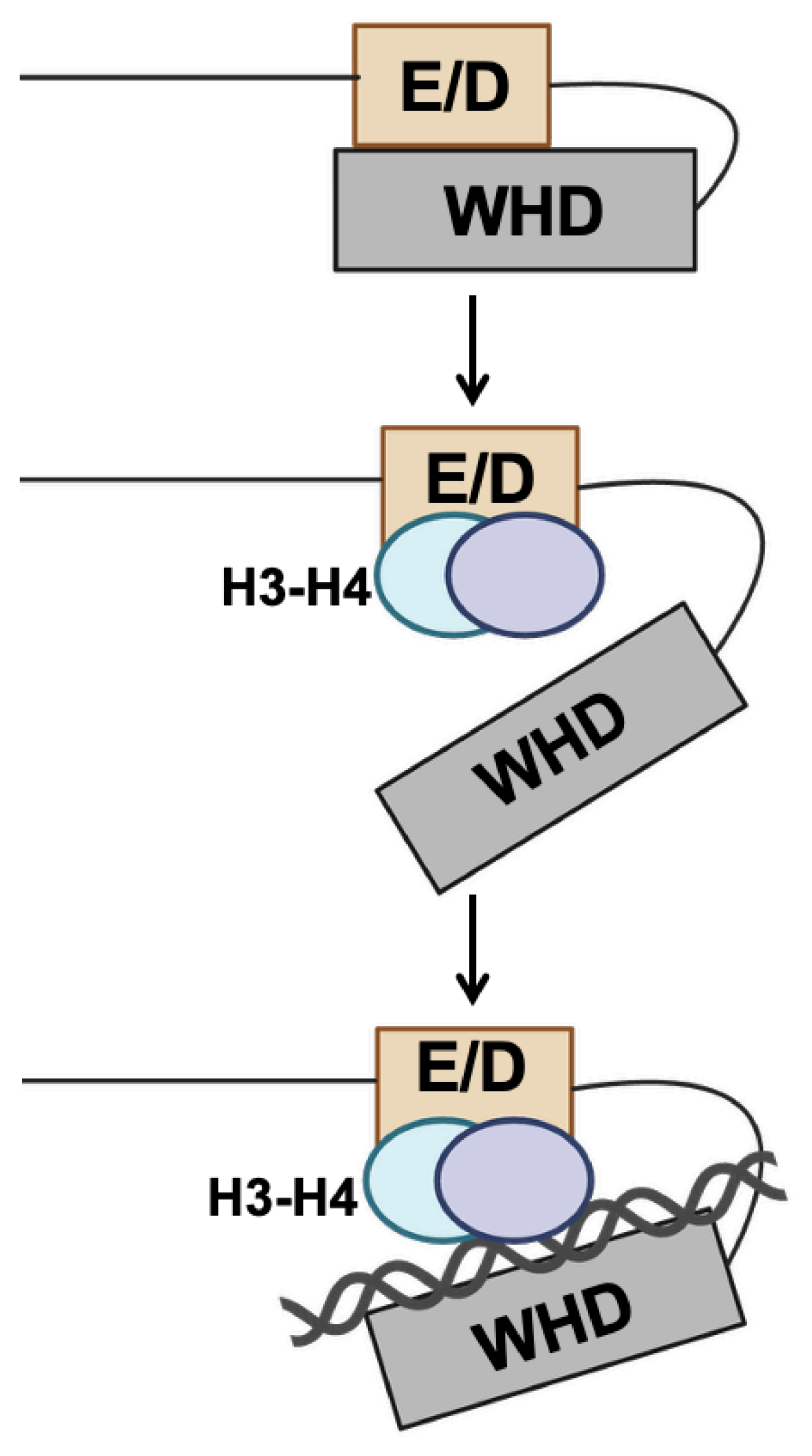
Model of the autoinhibition mechanism of histone- and DNA-binding in CAF-1. The E/D (tan), WHD (gray), and H3-H4 (blue/purple) histones are shown in orange, gray, and blue/purple, respectively. The rest of the Cac1 subunit is indicated by the black line.

WHD-mediated DNA binding is not straightforward but is instead subject to an additional layer of autoregulation. In the absence of DNA, the adjacent E/D domain forms an autoinhibitory interaction with the WHD, which must be relieved for productive DNA binding [15,45] (Figure 4). Because the E/D domain also contributes to histone binding, this autoinhibitory interaction must likewise be released before CAF-1 can recruit histones. Current evidence suggests that histone binding triggers this relief, initiating a cascade of conformational changes that enables the WHD to bind DNA [15,42]. Consequently, productive DNA binding is restricted to histone-loaded CAF-1 complexes. This mechanism would therefore promote efficient tetrasome assembly by preventing histone-free CAF-1 complexes from engaging DNA unproductively. Future studies are needed to explore the relationship between this model and the right-handed DNA-wrapping activity of CAF-1.

### 3.4. Middle and Small Subunits

The middle and small subunits of CAF-1 are both WD40-repeat proteins. Structurally, these are seven-bladed, β-propeller folds that associate to resemble a donut shape [44]. As discussed above, the WD40 of the middle subunit docks onto the MSI of the large subunit while simultaneously contributing directly to histone binding. The WD40 of the small subunit docks onto the SSI of the large subunit and makes minor contacts with histones [43]. Previous studies have suggested a major role for the small subunit in binding histones, where human p48 can bind to histone H4 in the absence of CAF-1 p150 and p60 [46]. However, other studies showed that deletion of the small subunit in yeast had only a moderate effect on H3-H4 binding and nucleosome assembly [43]. In contrast, deletion of the middle subunit resulted in near-complete loss of function [43]. It is possible that the small subunit contacts H3-H4 differently as an independent subunit than it does within the fully assembled CAF-1 complex. Alternatively, these differences may be context-dependent.

Importantly, the p48 subunit of human CAF-1 is known to exist in other chromatin-associated complexes, including the nucleosome remodeling and deacetylase complex (NuRD), and the polycomb repressive complex 2 (PRC2) [47,48]. In each complex, p48 acts as a scaffold for binding histones. This modularity is common in chromatin biology whereby a few small subunits or folds, such as WD40 repeats, are reused across many complexes to generate combinatorial and functional diversity [49,50]. Specificity of each complex typically comes from the large scaffolding subunit, such as the large subunit in CAF-1, that organizes these subunits into a complex with a specialized function.

## 4. Nucleosome Assembly

Nucleosome assembly occurs by functionally conserved mechanisms in humans and yeast. Following DNA replication and repair, nucleosomes are assembled with either nascent histones, synthesized in the cytosol and imported into the nucleus, or parental histones already present in the nucleus [51]. In either case, histones are passed through interconnected networks of chaperones before deposition onto DNA. Nascent and parental histones are distinguished by post-translational modifications (PTMs) [51,52]. However, the explicit molecular mechanisms by which PTMs affect histone–histone chaperone and histone–DNA interactions have yet to be fully elucidated. The assembly of nucleosomes following replication or repair begins with the transfer of two nascent or parental H3-H4 dimers from histone chaperones onto DNA and is completed by the addition of two flanking H2A-H2B dimers onto the periphery of the tetrasome. To highlight the role of CAF-1 in nucleosome assembly, the following discussion focuses on the transfer and deposition of H3-H4 rather than H2A-H2B.

### 4.1. Nascent H3-H4 Histone Chaperone Networks

**Figure 5:**
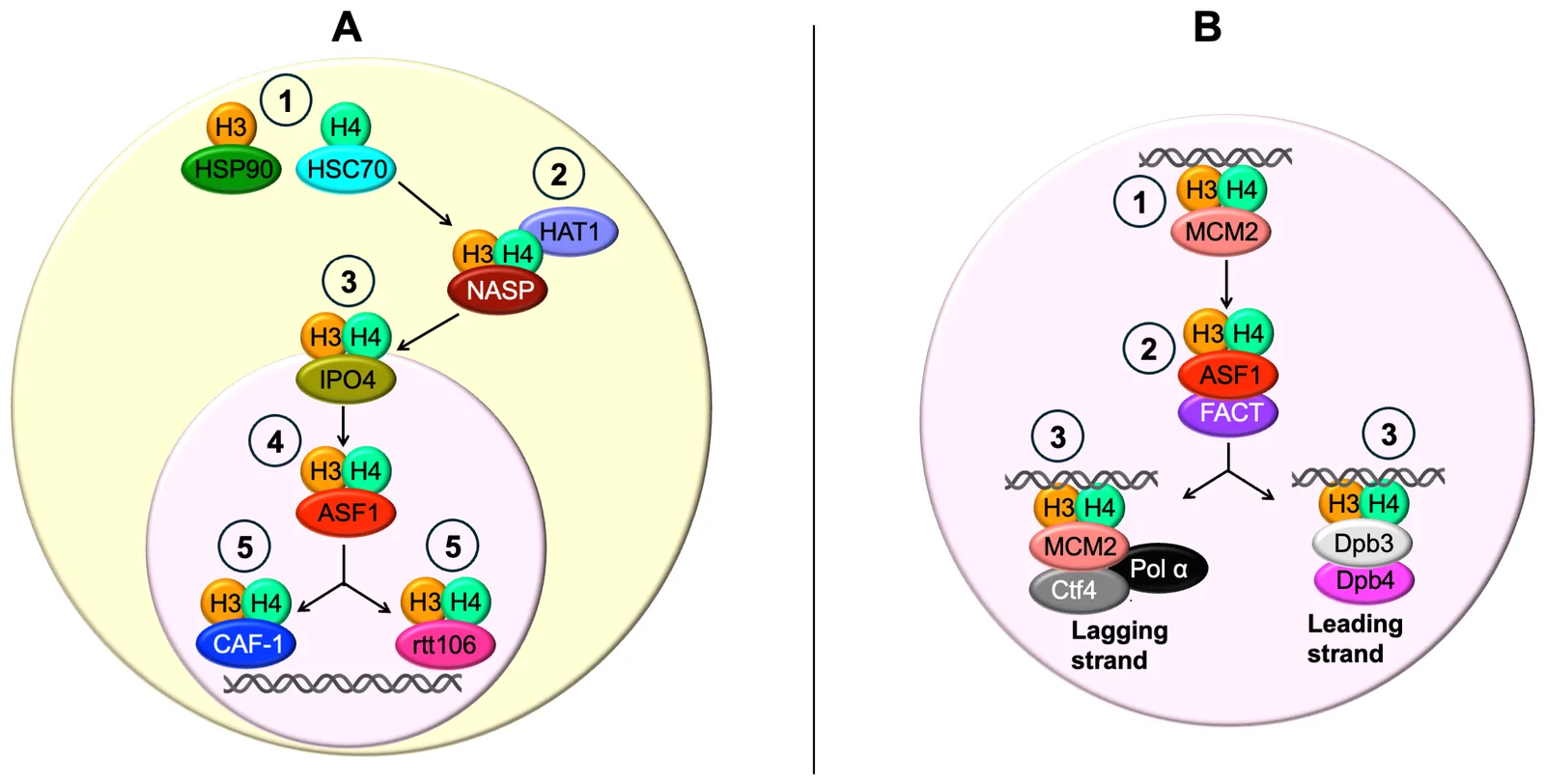
Overview of histone chaperone networks during nucleosome assembly. Schematic representation of the histone chaperone pathways regulating the transfer and deposition of (**A**) nascent and (**B**) parental histones. Sequential steps within each histone chaperone routing cascade are indicated numerically (circled numbers). The nucleus is denoted in pink.

The network of histone chaperones required for nascent histone transport pathways are summarized in Figure 5A. H3 and H4 histones are synthesized in the cytosol where heat shock cognate 70 (HSC70) and heat shock protein 90 (HSP90) facilitate proper folding [51,52]. Still in the cytosol, nuclear autoantigenic sperm protein (NASP) binds H3 and H4 histones and promotes formation of H3-H4 heterodimers [51]. NASP also recruits the histone-modifying enzyme histone acetyltransferase 1 (HAT1), which acetylates H4 on lysine 5 and 12 (H4K5/K12ac) [51,53]. H4K5/K12ac is an epigenetic mark that distinguishes nascent H3-H4 from parental H3-H4 [54]. Importin-4 (IPO4) transfers H3-H4 dimers from NASP in the cytosol to anti-silencing function 1 (ASF1) in the nucleus [55]. However, a mechanism in which H3 and H4 are imported as monomers by IPO4 has emerged as well [56]. In this mechanism, IPO4 transfers H3 and H4 monomers to NASP, located in the nucleus, which promotes the formation of the H3-H4 heterodimer. Ultimately, ASF1 binds nascent H3-H4 histones and transfers the histones to CAF-1 for deposition onto DNA [57]. ASF1 also binds parental H3-H4 during parental histone recycling; however, CAF-1 preferentially binds and receives H3-H4 histones marked with nascent histone PTMs from ASF1 [57]. In yeast, an additional histone chaperone, the regulator of Ty1 transposition protein 106 (rtt106), also receives nascent H3-H4 histones from ASF1 [58]. In yeast cells with deletion or mutation of CAF-1, RplNA is maintained by rtt106-mediated deposition of nascent H3-H4 onto DNA [58]. The rtt106 pathway represents an important yeast-specific backup mechanism for nascent H3-H4 deposition. After deposition, nascent H3-H4 are deacetylated and epigenetic marks for gene silencing or activation are installed by histone-modifying enzymes, thereby restoring the parental epigenetic state [59]. Parental H3-H4 dimers have several distinct methylation PTMs that promote the compaction of chromatin into heterochromatin or the relaxation of chromatin into euchromatin [59]. For instance, trimethylation of H3 on lysine 9 and 27 (H3K9me3 and H3K27me3) and tri/di-methylation of H4 on lysine 20 (H4K20me2/3) promote gene silencing through heterochromatin formation. In contrast, trimethylation of H3 on lysine 4 (H3K4me3) promotes a transcriptionally active chromatin state.

### 4.2. Parental H3-H4 Histone Chaperone Networks

CAF-1 preferentially binds nascent H3-H4 histones and deposits them onto DNA. Despite claims that CAF-1 also receives parental H3-H4 histones from ASF1 [60], CAF-1 is not widely believed to participate in the deposition of parental H3-H4 histones. The network of histone chaperones required for parental histone displacement and reassociation are summarized in Figure 5B. During RplNA, parental histones are transiently displaced from DNA ahead of the replication fork. Displaced H3-H4 tetramers are bound by minichromosome maintenance complex component 2 (MCM2) [61,62]. The MCM2-parental H3-H4 complex binds ASF1 and the parental histones are repositioned to interact with both MCM2 and ASF1 [62].

During recycling of parental H3-H4, ASF1, together with facilitates chromatin transcription (FACT), transfers parental histones to either DNA polymerase (pol) ε subunits Dpb3/Dpb4 or the MCM2/chromosome transmission fidelity 4 (Ctf4)/DNA polymerase α (Pol α) complex [63,64]. Dpb3/Dpb4 and MCM2/Ctf4/Pol α specialize in the deposition of H3-H4 onto the leading strand and lagging strand, respectively. In yeast, the histone chaperone rtt106 also receives parental H3-H4 histones from ASF1 and deposits them onto DNA [58]. A similar mechanism is presumed to occur during RprNA: parental histones are transiently displaced from DNA, transferred through histone chaperone networks and deposited onto DNA. However, molecular details for this mechanism remain to be determined.

### 4.3. The Role of CAF-1 in Replication-Coupled Nucleosome Assembly

To maintain epigenetic inheritance during RplNA, H3-H4 histones are deposited onto daughter DNA in a semi-conservative manner. Parental H3-H4 are re-deposited near the genomic loci they were displaced from and are distributed in nearly equal amount to both daughter DNA molecules [65]. However, the pool of parental histones is insufficient to recapitulate the parental epigenetic landscape onto the daughter DNA. Accordingly, nascent histones supplement nucleosome assembly following DNA replication. CAF-1 preferentially binds and deposits H3-H4 dimers that contain DNA synthesis-dependent variants of the H3 histone (H3.1 and H3.2) onto DNA [57,66]. H3.1 and H3.2 are constitutively expressed during S phase to supply the H3 histones required for RplNA [66]. H3.1 is expressed in both humans and yeast whereas the H3.2 variant is only expressed in humans and differs from H3.1 by a single amino acid substitution, C96S. A third H3 variant, H3.3, is often called the replacement variant or the synthesis-independent variant. H3.3 is expressed throughout the cell cycle and differs from H3.1 and H3.2 by five and four amino acids substitutions, respectively. H3.3 is deposited into nucleosomes during DNA synthesis-independent processes and is not known to interact with CAF-1 [57,67]. PCNA recruits CAF-1 loaded with H3.1/H3.2-H4 to the replication fork [8]. In a recently proposed model, two CAF-1–H3-H4 complexes associate with PCNA and together deposit an (H3-H4)_2_ tetramer onto the DNA to form the tetrasome [32,68]. H3 and H4 deposited onto DNA by CAF-1 typically contain acetylation modifications (such as H4K5ac, H4K12ac, or H3K9ac) that mark the nascent histones as substrates for histone-modifying enzymes that install parental PTMs and remove nascent PTMs after nucleosome assembly [54].

Recent biochemical studies suggest that CAF-1 deposits nascent H3-H4 histones onto the leading and lagging strands by distinct mechanisms during DNA replication. Like CAF-1, the leading strand DNA polymerase (Pol ε), and the lagging strand polymerase (Pol δ), contain PIP motifs and are recruited to the replication fork by PCNA. In primer extension assays, increasing concentrations of CAF-1 inhibited DNA synthesis by Pol ε, but not Pol δ, suggesting that CAF-1 may compete against Pol ε for PCNA binding [68]. The authors propose a model in which CAF-1 and Pol ε either bind separate PCNA trimers or CAF-1 and Pol ε dynamically exchange on and off the same PCNA trimer. However, in the absence of direct structural or displacement data, an alternative explanation remains plausible: CAF-1 may bind PCNA concurrently with Pol ε in a manner that directly or allosterically decreases Pol ε activity. In contrast, the presence of CAF-1 does not inhibit the activity of Pol δ bound to PCNA [68].

The insensitivity of Pol δ activity to CAF-1 in primer extension assays suggests that CAF-1 does not compete with Pol δ for PCNA binding [68]. Mechanistic differences underlying Pol ε and Pol δ function could help explain the asymmetry of CAF-1 function on leading and lagging strands. DNA is discontinuously synthesized on the lagging strand by Pol δ. After synthesis of an Okazaki fragment of approximately 150–200 bp in length, Pol δ dissociates from PCNA, leaving ample space for CAF-1 bearing H3-H4 histones to bind PCNA and initiate nucleosome assembly [69]. Conversely, Pol ε, which has lower affinity for PCNA, greater affinity for DNA, and greater processivity than Pol δ, synthesizes the leading strand continuously [70]. These properties of Pol δ are congruent with the proposed mechanism in which CAF-1 and Pol ε dynamically hand off PCNA during leading strand synthesis. In either scenario, the inhibitory effect of CAF-1 on Pol ε function likely slows leading strand synthesis to more closely match the rate of lagging strand synthesis.

### 4.4. The Role of CAF-1 in Repair-Coupled Nucleosome Assembly

DNA is damaged in eukaryotic cells by diverse mechanisms and at a staggering rate of around 10^4^ lesions per day [71]. To maintain genome integrity, DNA damage is addressed by several DNA repair mechanisms that can be broadly categorized as DNA synthesis-independent and DNA synthesis-dependent. Several small, non-helix-distorting lesions are repaired without DNA synthesis by direct enzymatic reversal or short-patch (1 nt) base excision repair (BER). Likewise, short-patch single-strand break repair (SSBR) and double-strand break repair (DSBR) by non-homologous end-joining (NHEJ) do not require DNA synthesis. Poly(ADP-ribose) polymerase 1 (PARP1) and PARP2 are among the first proteins to recognize single-strand breaks, binding the lesion and catalyzing poly(ADP-ribosyl)ation of chromatin and neighboring proteins [72,73]. This PARylation promotes the recruitment and coordination of downstream repair machinery that repairs short gaps [74,75]. Because synthesis-independent repair mechanisms do not require the passage of the replication machinery, nucleosomes largely remain in place during repair, and there is no need to couple nucleosome assembly to the repair process.

In contrast, the repair of bulkier, helix-distorting lesions and certain single-strand and double-strand DNA breaks requires the synthesis of new DNA under the coordination of PCNA. DNA synthesis-dependent DNA repair mechanisms include long-patch (2–10 nt) BER, nucleotide excision repair (NER), mismatch repair (MMR), long-patch SSBR, and DSBR by homologous recombination (HR) [76,77,78]. Notably, PARP1 activity can help dictate whether a DNA synthesis-dependent or -independent repair pathway is utilized. For instance, in BER, PARP1 interacts with and regulates the activity of polymerase β (Pol β). If the 5′-deoxyribose phosphate end generated after base excision is not removed by Pol β, repair is redirected from short-patch BER toward long-patch BER, in which PCNA-dependent strand-displacement synthesis by a replicative polymerase (Pol ε/δ) generates a short flap that is excised by FEN1 and ligated by DNA ligase I [79,80,81]. This polymerase switch effectively converts a synthesis-independent lesion into a synthesis-dependent one.

Synthesis-dependent mechanisms require nucleosomes to be removed from DNA to give the repair and synthesis machinery access to the site of damage. To preserve epigenetic inheritance, nucleosome assembly is tightly coupled to DNA synthesis-dependent repair mechanisms [16,82]. The removal and subsequent redeposition of H3-H4 histones during RprNA are presumed to proceed by a mechanism resembling RplNA: parental H3-H4 histones are transiently displaced from damaged DNA, handed off between histone chaperones, and then redeposited onto the repaired DNA. As in RplNA, CAF-1 is recruited by PCNA to deposit nascent H3-H4 histones onto the repaired DNA [8,16].

Specific PCNA mutations have been shown to selectively disrupt MMR [83], and structural analysis of these mutants revealed that subtle alterations in PCNA architecture—including changes in the central channel α-helices and a loop near the PCNA subunit interface—can impair productive interactions with MMR complexes [84]. Given that CAF-1 recruitment during repair depends on PCNA-mediated recognition at multiple sites on both proteins, these findings raise the possibility that PCNA conformational states may regulate the coordination between engagement of repair factors, replication factors, and CAF-1 during RprNA.

Multiple lines of evidence highlight the consequences of compromised CAF-1 function during RprNA. Without proper CAF-1 function, nucleosome assembly following NER is severely impaired, which prevents the re-establishment of chromatin structure [82]. Likewise, DSBs accumulate in human cells without functional CAF-1 [85,86]. Furthermore, a recent spatiotemporal profiling study demonstrated that in the absence of functional CAF-1, yeast cells displayed heterogenous delays in nucleosome maturation [85]. This finding suggests that nucleosome assembly in some regions of the genome can be recovered over time by backup mechanisms while others rely on CAF-1.

The deposition of H3-H4 histones during RprNA is less robustly characterized than during RplNA. Nonetheless, several mechanistic details for nucleosome assembly following NER and HR have emerged that challenge the presumptive model that CAF-1 exclusively deposits H3.1-H4 onto DNA repaired by synthesis-dependent pathways. During NER, bulky helix distorting lesions—such as those caused by UV radiation—are excised in approximately 30 nt patches, new DNA is synthesized to fill the resulting gap, and the DNA ends are ligated together [78]. However, NER has two sub pathways that share core excision and resynthesis mechanisms but differ in how lesions are recognized [78]. In most contexts, the global genome (GG) NER pathway repairs bulky UV-induced lesions. Following GG NER, CAF-1 is recruited to the site of DNA synthesis-dependent repair by PCNA to initiate nucleosome assembly by depositing nascent H3-H4 histones [16,82].

However, when bulky lesions are recognized during transcription, the transcription-coupled (TC) NER pathway instead repairs the damage [87,88]. During TC NER, bulky DNA lesions are recognized at stalled RNA polymerase II complexes [87,89]. Similarly to GG NER, the lesion is excised, and new DNA is synthesized under the coordination of PCNA [88,90]. However, before PCNA-mediated DNA synthesis begins, the H3-H4 chaperone histone cell-cycle regulator (HIRA) is recruited to the stalled transcription complex [91]. HIRA preferentially binds H3-H4 dimers that contain the synthesis-independent variant of H3, H3.3 [57]. Unlike CAF-1, HIRA does not contain a PIP motif and does not directly interact with PCNA. Instead, HIRA bound to H3.3-H4 is recruited to stalled transcription complexes early during TC NER [91]. The preemptive loading of the damaged DNA site with HIRA bound to H3.3-H4 effectively outcompetes the subsequent PCNA-mediated recruitment of CAF-1 coupled to DNA synthesis. Importantly, the HIRA-mediated deposition of H3.3-H4 onto the repaired DNA rather than the CAF-1-mediated deposition of H3.1/2-H4 promotes the formation of an accessible chromatin state that enables reinitiation of transcription [91].

Similarly to NER, the H3-H4 histone chaperone involved in RprNA following HR also depends on the context of the DNA repair. When a stalled or collapsed replication fork causes a double-strand break during S phase, HR repairs the DNA by synthesizing new DNA using the intact sister chromatid as a template and ligating the ends of the DNA together. PCNA coordinates DNA synthesis during HR and recruits CAF-1 to deposit nascent H3.1-H4 histones onto the repaired DNA, mirroring RplNA [78].

Recent work in budding yeast demonstrated that CAF-1 contributes to maintaining ribosomal DNA (rDNA) stability during replication-coupled DSBR [92]. Loss of CAF-1, or disruption of its interaction with PCNA, resulted in rDNA copy-number changes and production of extrachromosomal rDNA circles. CAF-1 deficiency also led to accumulation of resected DNA ends at the rDNA replication fork barrier and an increase in HR-dependent repair, suggesting that CAF-1-mediated chromatin assembly helps limit excessive DNA-end resection and aberrant HR at arrested replication forks.

However, double-strand breaks also occur outside of DNA replication during S phase. For example, endonucleases, ionizing radiation, and certain drug treatments cause double-strand breaks to DNA throughout the cell cycle. Mechanistically, the HR mechanism that repairs replication-independent double-strand breaks is the same; however, in humans, the histone chaperone death-domain-associated protein X (DAXX) has been reported to deposit dimers of H3.3-H4 onto the repaired DNA [93]. DAXX is better known for complexing with the chromatin remodeler alpha-thalassemia mental retardation X-linked (ATRX) to deposit H3.3-H4 onto DNA in repeat-rich regions of the genome like centromeres and telomeres [94,95]. However, recent work indicates that DAXX-dependent H3.3 loading at double-strand breaks can occur independently of ATRX [93]. The role of ATRX in RprNA following HR, if any, remains to be resolved. Because replication-independent HR requires DNA synthesis facilitated by PCNA, CAF-1 is presumed to be recruited to the repaired DNA by PCNA. However, the interplay between CAF-1-mediated and DAXX-mediated H3-H4 deposition requires further research to disentangle.

The interaction between CAF-1 and PCNA mediates the deposition of H3-H4 histones in most DNA synthesis-dependent process like DNA replication and synthesis-dependent DNA repair [8,16]. However, context-specific exceptions to the presumptive CAF-1–PCNA-mediated model of H3.1/2-H4 deposition including HIRA- and DAXX-mediated deposition of H3.3-H4 have emerged [91,93]. Further research is required to parse how and when alternative non-CAF-1–PCNA-mediated mechanisms are implemented to deposit H3-H4 histones during RprNA.

### 4.5. CAF-1 Functions Beyond Replication and Repair

Although CAF-1 is best characterized as the histone chaperone that couples nucleosome assembly to DNA synthesis during RplNA and RprNA, an expanding body of work indicates that this same activity has consequences that extend beyond the immediate restoration of chromatin behind the replication fork or at a repaired lesion. CAF-1 also participates in establishing facultative heterochromatin, the developmentally regulated chromatin state marked by H3K27me3 that silences lineage-inappropriate genes as cells differentiate. Deletion of the p150 subunit in mouse embryonic stem cells (ESCs) impairs differentiation and disrupts silencing of core pluripotency genes [96]. Notably, p150 appears dispensable for maintaining H3K27me3 that is already in place. This indicates CAF-1 is not required to maintain pre-existing facultative heterochromatin but is instead required to newly establish it during a developmental transition. CAF-1 activity also restrains development at the other side of differentiation. Reducing CAF-1 activity caused a rare subpopulation of mouse ESCs to revert into a flexible, early-embryo state that is much easier to reprogram [97]. This demonstrates the role of CAF-1 in depositing histones is to act as an “epigenetic memory” that locks a cell’s identity into place during division.

Importantly, CAF-1 is essential for viability in many organisms. Loss of CAF-1 is lethal in every multicellular animal model examined to date, in contrast to budding yeast, where CAF-1 deletion is fully viable with only silencing and DNA-damage-sensitivity defects. Deletion of the mouse p150 subunit is embryonically lethal: homozygous mutant embryos arrest around the 16-cell stage with severely disorganized constitutive heterochromatin [98]. In *Drosophila*, null mutants of the large subunit are hemizygous lethal, block the ability of the female to produce eggs, and disrupt eye development [99]. Together, these studies suggest CAF-1 activity becomes more significant as an organism grows more complex. In single-celled yeast, loss of CAF-1 causes more minor, local gene-regulation errors. However, in multicellular animals, CAF-1 is essential for survival because it is required to accurately lock cell identity into place as cells divide and form complex tissues.

## 5. Conclusions

Recent structural and biochemical studies have uncovered increasingly sophisticated mechanisms underlying PCNA-mediated CAF-1 recruitment to DNA, involving coordinated protein–protein interactions, DNA-binding domains, and regulatory elements that promote efficient nucleosome assembly during DNA replication and repair. Together, the three CAF-1 subunits function cooperatively to couple DNA synthesis with chromatin assembly. PCNA recruits CAF-1 to replication forks and sites of DNA repair, where the largest subunit positions the complex on the DNA, the largest and middle subunits coordinate the transfer of newly synthesized H3-H4 from ASF1, and the small subunit stabilizes histone binding during deposition. CAF-1 subsequently promotes assembly of newly synthesized H3-H4 into (H3-H4)_2_ tetramers on DNA, creating the foundation for H2A-H2B incorporation and complete nucleosome formation. Through this coordinated mechanism, CAF-1 restores chromatin following DNA replication and repair, thereby preserving epigenetic information and genome stability.

Despite considerable advances, the molecular mechanism by which CAF-1 coordinates interactions with PCNA, DNA, and histones during nucleosome assembly remains to be elucidated. One important unanswered question is why two PIP motifs exist in CAF-1 and the functional relationship between them. In addition, the reason for large discrepancies between the structure, binding kinetics, and function of these motifs in human and yeast remains unclear. Whether CAF-1 binds multiple PCNA subunits simultaneously, transitions between conformationally distinct PCNA-binding states, or dynamically exchanges with other PCNA-binding proteins at replication forks remains unknown. These questions are particularly important, as CAF-1 must function in an environment crowded with numerous PCNA-binding proteins, including DNA polymerases, repair factors, and cell-cycle regulators, all competing for access to the same sliding clamp.

The mechanisms governing histone-specific binding and deposition by CAF-1 and the complex interplay between domains within the CAF-1 complex are also unresolved. Although CAF-1 is generally considered a chaperone for newly synthesized histones, several studies suggest it may interact with recycled parental H3-H4. The precise functions of the CAF-1 DNA-binding domains during nucleosome assembly also remain to be determined. Although both the K/E/R domain and the WHD bind DNA, their individual contributions appear partially redundant and differ among species. Furthermore, the acidic E/D domain not only binds histones but also autoinhibits DNA binding by the WHD until histones have been loaded, suggesting that CAF-1 undergoes a series of coordinated conformational changes during nucleosome assembly. It is unknown how these conformational transitions are triggered and how they are coordinated with CAF-1–PCNA and CAF-1–histone interactions remains to be determined. It will be important to determine whether the presence of DNA strengthens these interactions. Finally, while the structure of human CAF-1 has been determined, there exists no high-resolution structure of the heterotrimeric yeast complex. This structure would aid in our understanding of how the three CAF-1 subunits cooperate structurally throughout replication- and repair-coupled nucleosome assembly. And although replication-coupled nucleosome assembly is comparatively well characterized, repair-coupled nucleosome assembly appears to be more context-dependent.

Collectively, this review highlights the fact that nucleosome assembly is governed by an interconnected network of dynamic protein–protein, protein–histone, and protein–DNA interactions. Accurate and efficient coordination of multiple CAF-1 mechanisms, including recruitment by PCNA, histone chaperone exchange, binding of DNA, conformational regulation within CAF-1, and histone variant selection is necessary for proper epigenetic inheritance. Future work will help further define how these individual processes are temporally and spatially coordinated during replication and DNA repair.

## Data Availability

No new data were created or analyzed in this study. Data sharing is not applicable to this article.

## Supplementary Materials

The following supporting information can be downloaded at: https://www.mdpi.com/article/10.3390/biom16091277/s1, Figure S1: Intermolecular interactions of the protein-protein interaction interface between the PIP1 motif of *S.c.* CAF-1 and PCNA; Figure S2: Intermolecular interactions of the protein-protein interaction interface between the PIP2 motif of *S.c.* CAF-1 and PCNA (PDB: 8THW).

## Abbreviations

The following abbreviations are used in this manuscript:

PCNA	Proliferating cell nuclear antigen
CAF-1	Chromatin assembly factor 1
PIP	PCNA-interacting protein
RplNA	Replication-coupled nucleosome assembly
RprNA	Repair-coupled nucleosome assembly
IDCL	Interdomain connecting loop
SIM	SUMO-interacting motif
HP1	Heterochromatin protein 1
SSI	Small subunit-interacting
MSI	Middle subunit-interacting
WHD	Winged helix domain
NuRD	Nucleosome remodeling and deacetylase complex
PRC2	Polycomb repressive complex 2
PTMs	Post-translational modifications
HSC70	Heat shock cognate 70
HSP90	Heat shock protein 90
NASP	Nuclear autoantigenic sperm protein
HAT1	Histone acetyltransferase 1
IPO4	Importin-4
ASF1	Anti-silencing function 1
rtt106	Regulator of Ty1 transposition protein 106
MCM2	Minichromosome maintenance complex component 2
FACT	Facilitates chromatin transcription
Ctf4	Chromosome transmission fidelity 4
Pol α	DNA polymerase α
Pol ε	DNA polymerase ε
Pol δ	DNA polymerase δ
Pol β	DNA polymerase β
BER	Base excision repair
SSBR	Short-patch single-strand break repair
DSBR	Double-strand break repair
NHEJ	Non-homologous end-joining
NER	Nucleotide excision repair
MMR	Mismatch repair
HR	Homologous recombination
GG	Global genome
TC	Transcription coupled
HIRA	Histone cell-cycle regulator
DAXX	Death-domain-associated protein X
ATRX PARP	Alpha-thalassemia mental retardation X-linked Poly(ADP-ribose) polymerase
