# Supplementary material for: CAF-1 in Replication and Repair: A Critical Genetic Mediator of Synthesis-Coupled Nucleosome Assembly
Source: Biomolecules. 2026 Sep 3;16(9):1277. doi: 10.3390/biom16091277 (PMC13604520; doi:10.3390/biom16091277)
Supplement: Supplementary file 1 [file biomolecules-16-01277-s001.zip › biomolecules-4485855-supplementary.pdf]

Supplementary Figures

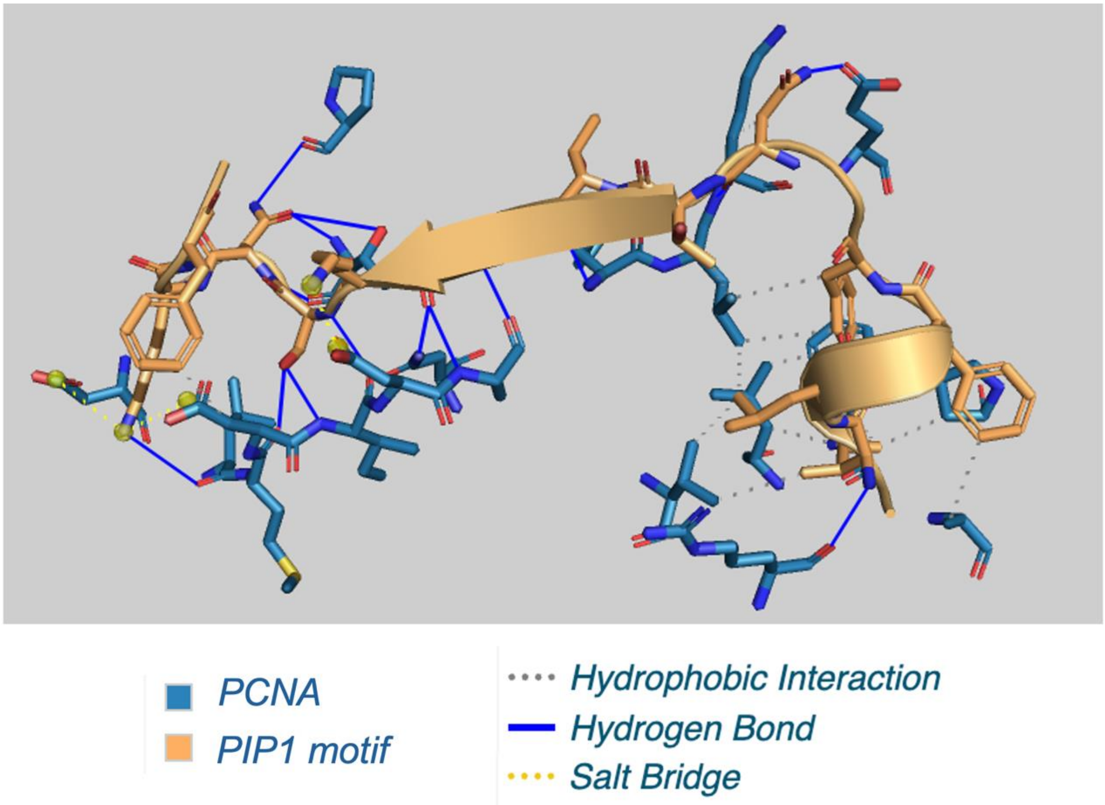

Figure S1. Intermolecular interactions of the protein–protein interaction interface between the PIP1 motif of *S.c.* CAF-1 and PCNA. A 3D molecular representation showing the specific binding interactions between the PIP1 motif (light orange ribbon/sticks) and PCNA (blue sticks). Non-covalent interactions are delineated as follows: gray dotted lines indicate hydrophobic interactions; solid blue lines represent hydrogen bonds; yellow dotted lines depict salt bridges. Oxygen and nitrogen atoms are colored red and dark blue, respectively. (Structure from: Hall, I., Davies, I.M., Limaye, S.A., Williams, I.L., Carlson, C.E., Snetsinger, T.J., Agbakpo, A.E., Checco, J.W., and Dieckman, L.M. A Second, Extended PIP Motif in Yeast CAF-1 Mediates Multivalent Recognition by PCNA. *Biomolecules* 2026, submitted)

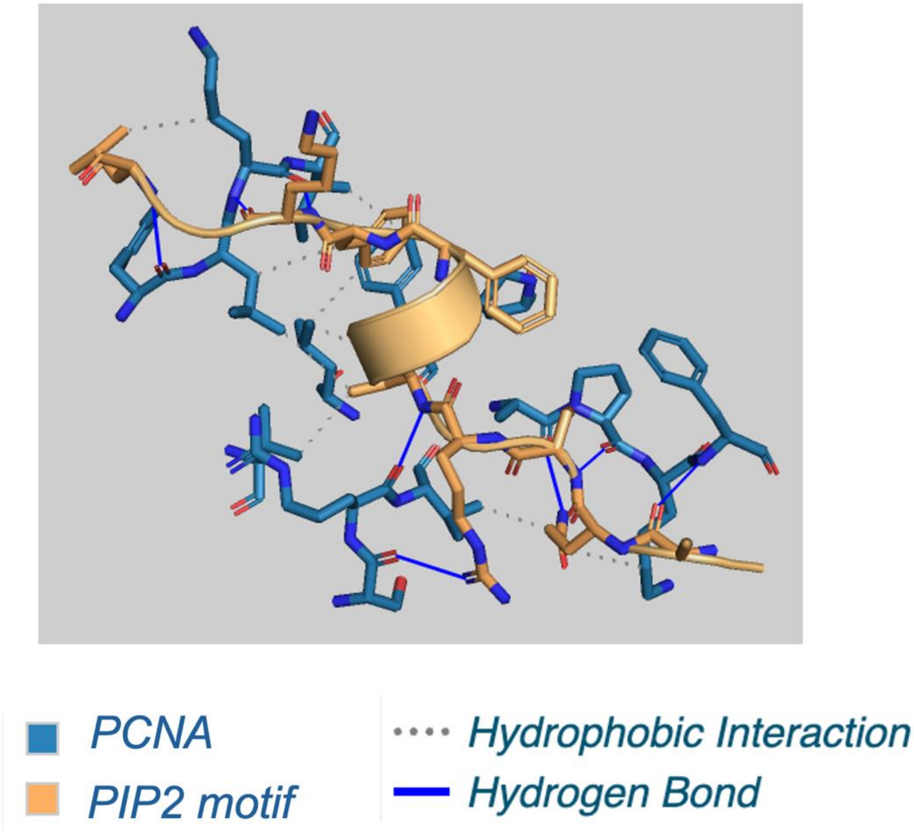

Figure S2. Intermolecular interactions of the protein–protein interaction interface between the PIP2 motif of *S.c.* CAF-1 and PCNA (PDB: 8THW). A 3D molecular representation showing the specific binding interactions between the PIP2 motif (light orange ribbon/sticks) and PCNA (blue sticks). Non-covalent interactions are delineated as follows: gray dotted lines indicate hydrophobic interactions and solid blue lines represent hydrogen bonds. Oxygen and nitrogen atoms are colored red and dark blue, respectively.
